# Dynamic adaptive X-ray optics. Part I. Time-resolved optical metrology investigation of the bending behaviour of piezoelectric bimorph deformable X-ray mirrors

**DOI:** 10.1107/S1600577518015953

**Published:** 2019-01-01

**Authors:** Simon G. Alcock, Ioana-Theodora Nistea, Riccardo Signorato, Kawal Sawhney

**Affiliations:** a Diamond Light Source, Harwell Science and Innovation Campus, Didcot, Oxfordshire OX11 0DE, UK; b S.RI. Tech s.r.l.s, Viale del Lavoro 42A, 35010 Vigonza, Padova, Italy

**Keywords:** adaptive X-ray optics, piezoelectric bimorph deformable mirrors, Fizeau interferometry, high-voltage power supplies, strain-free holders

## Abstract

Piezoelectric bimorph deformable mirrors (‘bimorphs’) are routinely used on many synchrotron and free-electron laser beamlines to provide active variation in the size and shape of the X-ray beam. However, the time-domain potential of such optics has never been fully exploited. For the first time, the fast dynamic bending response of bimorphs is investigated here using Fizeau interferometry. Automated scripts for acquisition and analysis were developed to collect Fizeau data at a rate of 0.1 Hz to record dynamic changes in the optical surface as voltages were applied to the electrodes of the piezoelectric actuators. It is demonstrated that residual drift in the tangential radius of curvature of a bimorph can be significantly reduced using enhanced opto-mechanical holders and a fast programmable high-voltage power supply. Further improvements are achieved by applying small opposing voltages to compensate for piezoelectric creep. The present study shows that bimorphs can truly be used as high-speed adaptive optics for the X-ray domain, even without closed-loop feedback correction. This opens the possibility for relatively simple real-time tuning of the profile of X-ray bimorphs. Part II of this study [Alcock, Nistea, Signorato, Owen, Axford, Sutter, Foster & Sawhney (2019), *J. Synchrotron Rad.* **26**, 45–51] builds upon these results and demonstrates how bimorphs can rapidly provide customisable sizes and shapes of synchrotron X-ray beams, specifically tailored to suit the experimental samples being investigated.

## Abstract 2

The time-domain bending behaviour of piezoelectric bimorph deformable X-ray mirrors for use at synchrotron and X-ray free-electron laser sources is investigated for the first time using high-speed Fizeau interferometry. It is demonstrated that several hardware and software innovations enable the optical surface of such mirrors to be rapidly tuned and stabilized on the nanometre scale within only a few seconds.

## 1. Introduction   

Since their initial development for X-ray applications in the mid-1990s (Susini *et al.*, 1996 ▸; Signorato *et al.*, 1998 ▸), piezoelectric bimorph deformable mirrors (‘bimorphs’) have been widely used at many synchrotron radiation and X-ray free-electron laser (XFEL) facilities to focus X-ray beams. Such bimorphs have been commercially available for two decades from Thales-SESO (TSESO), France. Currently, 19 bimorphs, up to a metre in length, are deployed on nine beamlines at Diamond Light Source (Diamond), UK. Multiple piezoelectric ceramic blocks are either embedded between polished optical substrates (first-generation bimorphs; Signorato *et al.*, 1998 ▸; Alcock *et al.*, 2013 ▸) or bonded to the side faces (second-generation bimorphs; Alcock *et al.*, 2015 ▸). Conductive metal electrodes, deposited onto the ceramics, form discrete piezo actuators (typically between 8 and 32 per mirror). The mirror substrate bends when voltages are applied to the piezo ceramics via each electrode. Aside from accurately bending the silicon or fused silica substrate to a range of curvatures to suit a variety of focusing geometries of the beamline, the individual piezoelectric actuators also enable localized zonal control of the mirror’s surface.

Due to the short wavelength of X-rays, the biggest challenge for state-of-the-art X-ray mirrors is achieving sub-nanometre surface height (figure) errors. Recent innovations in deterministic ‘super-polishing’ techniques, including ion-beam figuring (IBF) (Hänsel *et al.*, 2004 ▸) and elastic emission machining (EEM) (Takei *et al.*, 2013 ▸), have significantly improved upon traditional mechanical polishing methods. Substrates with slope errors <200 nrad r.m.s. can now be routinely fabricated by several vendors. Coupled with the accurate and highly resolved bending capabilities provided by bimorphs, such substrates are capable of exceptional focusing. They can also reshape the X-ray beam and correct wavefront aberrations introduced by non-ideal optics elsewhere on the beamline (Sawhney *et al.*, 2013 ▸).

Numerous metrology studies at Diamond, and anecdotal evidence from other metrology laboratories, have shown that older bimorph mirrors typically take several minutes to change shape after a large voltage change. It is therefore prudent to wait at least 15 min after applying a large voltage change before correcting the surface profile using inverse-matrix methods and piezo response functions (PRF). Accurate PRFs ensure that only a single iteration of voltage correction is typically required to optimize a bimorph to a given surface profile using feedback from visible light or X-ray metrology. However, the mirror’s curvature can continue to drift on a small scale for many hours afterwards. It was hypothesized that such drifts were caused by mechanical holders and stiff electrical connectors that were resisting the action of piezo bending. This accumulated elastic energy is either gradually released over several hours or days in a continuous manner or via a series of discrete steps through stick/slip frictional behaviour. This leads to a small relaxation of the mirror’s curvature, typically by a few percent, but in extreme cases by up to 10% (Alcock *et al.*, 2015 ▸). In some instances, dictated by the beamline optical layout, such curvature drifts may cause noticeable variations in the size, shape or position of the focused X-ray beam. In the past, beamline users tolerated such drifts rather than continuously retuning the mirrors to achieve ultimate stability. However, small changes in the size of the focused X-ray beam, that were once hidden by the intrinsic blurring caused by optical slope errors, are now becoming increasingly evident due to the sharper focus provided by super-polished substrates. Once such X-ray beam drifts become routinely observable, this motivates the study of their time-domain behaviour to mitigate the risk of reduced beamline performance.

To exploit the operational potential of bimorphs fully, it is therefore important to understand and quantify the practical factors that influence the time dependence of their bending action. Until now, to the best of the authors’ knowledge, all aspects related to the dynamic use of bimorphs have largely been neglected. Due to their excellent long-term stability, X-ray mirrors were typically tuned to a specific shape and then left untouched for days, if not weeks or months. However, as beamlines strive for ever faster experiments and greater flexibility in X-ray beam control, the temporal domain becomes increasingly important. Therefore, to enable quasi-continuous adaptive tuning of X-ray beams, significant effort and resources have recently been invested by the Optics and Metrology group at Diamond to improve the mechanical and electrical hardware for bimorphs and their control software. Our study concentrates on the validation of these innovations and improvements.

For the first time, using ex situ metrology feedback from a Fizeau interferometer, we investigate the dynamic evolution of the optical surface of several microfocusing bimorphs and determine whether they can be driven at higher speeds without causing residual curvature drifts. Excellent agreement is typically achieved between ex situ measurement of active X-ray optics and in situ X-ray characterization (Sutter *et al.*, 2012 ▸). This provides confidence that lessons learnt using visible-light metrology, such as slope profilometry or interferometry, can provide a reliable assessment of how optics will perform with X-rays on the beamline. In the follow-up paper (Part II; Alcock *et al.*, 2019 ▸) we build upon these results to present a proof-of-principle beamline experiment showing how microfocus bimorphs are capable of high-speed control of the size and shape of a synchrotron X-ray beam.

## 2. Experimental   

### 2.1. Piezoelectric bimorph deformable mirrors   

A comparative study was performed in the Optical Metrology Laboratory at Diamond (Alcock, Nistea & Sawnhey, 2016 ▸) to understand and quantify the factors which influence the dynamic bending behaviour of microfocusing bimorph mirrors. Three representative mirrors were selected to represent a broad historic range of substrate and holder types, from an old and obsolete bimorph to the newest current design. This also provides a guide to the evolution of such optics over the past 15 years. As listed in Table 1 ▸, all mirrors are either first- or second-generation bimorphs manufactured by TSESO, are mounted in either older non-kinematic (TSESO) or new kinematic (S.RI. Tech) holders, and have either rigid electrical connectors or flexible wires to apply voltages to the electrodes. For the sake of consistency, all substrates are of similar dimensions and made from fused silica. Mirrors 1 and 3 have metal coatings for enhanced X-ray reflectivity, whereas Mirror 2 is uncoated.

Aside from their symmetric construction, which makes them rather insensitive to changes in ambient temperature, one of the major advantages of bimorphs compared with mechanical benders is that bending is achieved by means of internal forces. Moreover, bimorphs do not dissipate power during bending and can hold a given shape indefinitely. Hence, it is theoretically possible to drive them continuously between different shapes, in a very repeatable way, for extended periods of time and without generating heat. Three main factors contribute to the time taken for a bimorph to stabilize dynamically to a new tangential curvature: (i) time to apply voltages; (ii) mechanical drift induced by the opto-mechanical holder; and (iii) intrinsic creep of the piezoelectric ceramics. Can such influences be quantified and better controlled, eventually leading to bimorphs with almost instantaneous response and improved stability? If so, this enables the time-domain capabilities of bimorphs to be exploited, thereby progressing beyond the current usage of active X-ray optics in a quasi-static ‘set and forget’ mode.

### 2.2. Voltage application and stabilization   

Bipolar high-voltage (HV) power supplies, optimized for bimorphs, are commercially available from a number of suppliers. Ideally, the combination of power supply and bimorphs should be sufficiently repeatable and stable to be driven according to a simple look-up table of voltages, without the need for continuous monitoring and feedback. This avoids the complexity and cost associated with implementing and operating sophisticated beamline optical monitoring systems for closed-loop feedback of the mirror’s shape. At Diamond, high voltages (±2000 V) are supplied to several of the bimorph mirrors using an SY900S or MAS-TER high voltage power supply from Sincrotrone Trieste/ELETTRA, Italy. These systems operate in three modes, including a fast mode in which the mirror is driven with relatively short stabilization times, but still takes several minutes to reach its final target shape. Electrical tests have routinely shown that these power supply units provide stable and repeatable high voltage to the bimorphs. However, their complexity of use and restricted functionality hinder scientific usage if speed is an issue. A new HV power supply unit, HV-ADAPTOS (based on the ELETTRA design), has recently become commercially available. It is manufactured by CAEN and distributed by S.RI. Tech. This device has enhanced on-board computational power and is readily programmable, enabling the user rapidly to change and optimize the parameters of the applied voltage profile, including variable slew rates and correction of hysteresis and piezo creep. The HV-ADAPTOS power supply was programmed to operate in two modes: high accuracy, with a slow slew rate <10 V s^−1^ to mimic the settings used by the former SY900S power supply from ELETTRA, and a new fast configuration with a slew rate >50 V s^−1^. The high-accuracy mode deliberately applies a damped sinusoidal voltage oscillation after the main impulse.

### 2.3. Mechanically induced drift   

**Figure 1:**
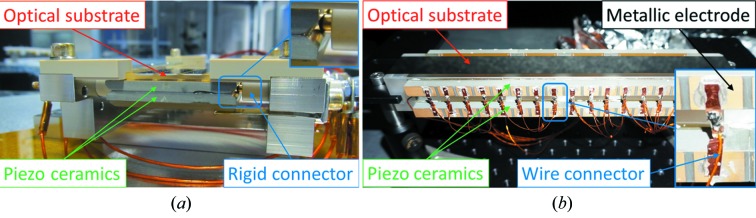
Earlier metrology studies indicated that the rigid electrical connectors [panel (*a*)], which originally supplied high voltage to the piezo ceramics, were resisting the bending of bimorph deformable X-ray mirrors. To correct this issue, TSESO now routinely use flexible wire connectors [panel (*b*)] on more recent bimorphs.

**Figure 2:**
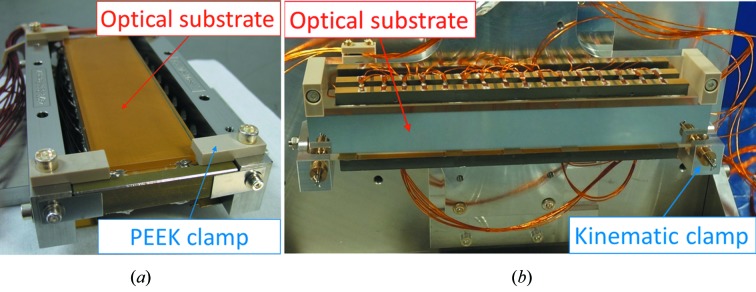
Older microfocus bimorphs were typically secured in opto-mechanical holders using PEEK clamps [panel (*a*), showing Mirror 2 from Table 1 ▸]. The non-kinematic nature and flexibility of the PEEK clamps meant that the curvature of such mirrors often drifted by a few percent over several hours. To improve bending stability, a three-point spring-loaded kinematic clamping system was developed by S.RI. Tech [panel (*b*), showing Mirror 3].

Even when resting unmounted and unconstrained, a bimorph substrate will bend deterministically when voltages are applied to its electrodes. Hence, it is only necessary to build a holder that ensures a safe and rigid connection between the positioning mechanics and the optical substrate. Ideally, a perfect holder should be intrinsically transparent to the bending action generated within the bimorph mirror itself. However, this requirement is contrary to the strong and stiff connection that is typically used, whereby the optic is firmly clamped into its holder after manufacture at TSESO. This approach mitigates the risk of damage during international transport and beamline installation. Over the past few years, two methods have been employed to reduce the influence of the opto-mechanical holder from distorting the mirror. Firstly, as seen in Fig. 1 ▸, the rigid electrical connectors applying voltages to the piezos were replaced with flexible wires by TSESO. In Fig. 1 ▸(*a*), the enlarged region shows that each rigid electrical connector is mechanically pressed against the two piezo ceramic plates (separated by a thin earthing layer) of a first-generation bimorph. In Fig. 1 ▸(*b*), the enlarged region shows a thin metal strip bonded to the discrete metallic electrodes on the upper and lower piezo ceramic bars of a second-generation bimorph. A flexible kapton-coated wire connector is bonded to each of these thin metal strips to provide the same voltage to the corresponding electrodes on the upper and lower piezo ceramic bars. Since the flexible wires cannot mechanically counteract changes to piezo bending, this should reduce the magnitude of the drift. To further reduce strain imparted to the substrate by the holder, the second innovation consists of replacing the original non-kinematic clamps made from polyether ether ketone (PEEK) shown in Fig. 2 ▸(*a*). The PEEK plastic clamps do not provide a very stable surface against which to press and hold the substrate. A novel opto-mechanical holder with a three-point kinematic interface was recently designed by S.RI. Tech and built by CINEL, Italy, for a 550 mm long second-generation bimorph mirror at Diamond. The new holder was deliberately designed such that the holder and substrate are transported separately, which strongly reduces the need for rigid shock-proof clamping. Once delivered to Diamond, the mirror was gently secured into its holder using three spring-loaded kinematic clamps. Metrology tests showed that the holder retained the bimorph mirror securely, without over-constraining it or adding angular vibrations. After this success, similar holders were designed and built for Mirror 3 [Fig. 2 ▸(*b*)] and its Kirkpatrick–Baez (KB) partner.

### 2.4. Creep of piezoelectric actuators   

All piezoelectric devices exhibit hysteresis and creep (Jung & Gweon, 2000 ▸), the temporal rate and magnitude of which are influenced by, amongst other factors, the recent history of applied voltages. Specifically, piezoelectric ceramics expand logarithmically in time due to creep (Vieira, 1986 ▸). Since piezos expand linearly in response to an applied voltage, and the inverse of the radius of curvature is linearly proportional to the applied voltage (Susini *et al.*, 1996 ▸), we expect that the inverse radius of curvature of a bimorph will drift logarithmically in time due to piezoelectric creep. Importantly, creep occurs in the same direction as dimensional changes in the piezoelectric ceramic induced by the applied voltage. For example, a positive voltage change will expand the piezo ceramics and flatten a concave mirror. The mirror’s surface then continues to flatten logarithmically in time. It is not straightforward to calculate explicitly how efficiently piezo creep is transferred to the bimorph substrate, as this depends precisely on multiple parameters, including the dimensions and material of the substrate (silicon or glass) and the geometry and location of piezoelectric actuators. It is important to note that second-generation bimorphs (with piezoelectric ceramic bars glued to the sides of the substrate) are likely to behave slightly differently from first-generation bimorphs (piezo ceramics glued in a sandwich between optical substrates). For example, the ratio between the volume of the substrate and the piezo ceramics is typically much larger for second-generation bimorphs. Additionally, piezo forces are applied indirectly from the sides for second-generation bimorphs, rather than directly from below as with first-generation bimorphs. Such arguments mean that the influence of piezo creep for second-generation bimorphs is likely to be damped, but at the cost of a slightly reduced bending range. However, it is a misconception that second-generation bimorphs have a limited bending range: one simply needs to make the substrate slightly thinner to achieve the same bending range as a comparable first-generation bimorph.

### 2.5. Fizeau interferometry   

Since 2007, the Diamond-NOM slope profilometer (Alcock *et al.*, 2010 ▸) has provided valuable metrology information about X-ray optical systems, including the bending behaviour of active optics. Although this slope profiler is capable of measuring planar or curved X-ray optics with a repeatability of <50 nrad r.m.s., it takes at least 3 min to perform each step scan. Therefore, despite being capable of monitoring drifts over many hours, it does not have sufficient temporal sensitivity to investigate rapid changes in the surface of the bimorphs caused by applying voltages to the piezos. A new approach was necessary to achieve the goal of characterizing the dynamic bending behaviour of bimorphs on timescales shorter than 1 min.

**Figure 3:**
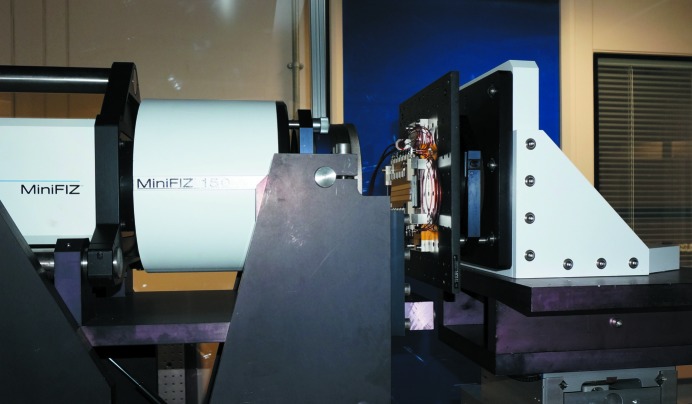
MiniFiz150 Fizeau interferometer for the rapid capture of dynamic changes in the tangential curvature of a bimorph deformable mirror (Mirror 1 in Table 1 ▸) in response to a range of large voltage impulses. Topography maps of the optical surface were captured every 10 s for >1 h for each experimental configuration.

To improve the acquisition rate significantly, we investigated the effectiveness of using a MiniFiz150 Fizeau laser interferometer manufactured by ADE Phaseshift (Fig. 3 ▸). Operating in single-pass mode, the surface normal of the optic to be tested is aligned anti-parallel to the Fizeau’s 150 mm-diameter laser beam. Individual phase-shifted topography maps of the surface under test are obtained roughly every 2.5 s. Proof-of-principle tests showed sub-nanometre noise levels could be achieved by averaging four successive scans, resulting in the acquisition of high-quality topography maps every 10 s. Although the random noise levels of the Fizeau are higher than those of the Diamond-NOM, the acquisition speed is enhanced by a factor of ∼20, thus allowing the temporal behaviour of the bimorphs to be studied on significantly quicker timescales.

Each Fizeau topography map, relative to the high-quality λ/200 (peak-to-valley) transmission flat, contains more than 350k pixels. With six images collected per minute, for tens or even hundreds of minutes, significant volumes of data are generated. For multiple voltage changes, this leads to tens of thousands of topography images (tens of gigabytes) to be processed. To analyse such quantities of data, MATLAB scripts were written to batch process hundreds of data sets and automatically compute important optical parameters such as the tangential radius of curvature as a function of time. For each image, the best-fit cylindrical radius of curvature was calculated from a central tangential line, 100 mm long, and averaged in the sagittal direction over 9 pixels (corresponding to ∼3 mm). When measuring any of the mirrors in a steady state (*i.e.* many hours since the last voltage change), the r.m.s. deviation in the measurement of the radius of curvature was typically only ∼0.01% relative to its average value.

## 3. Results   

**Figure 4:**
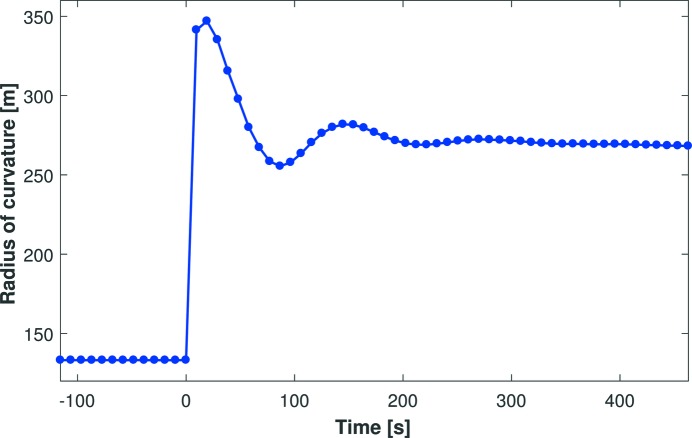
The dynamic radius of curvature of a bimorph deformable mirror as a function of time after applying a 1000 V change to all piezos, followed by damped voltage oscillations. Over each 10 s interval, the Fizeau interferometer recorded the mirror’s radius of curvature with sufficient sensitivity to resolve all dynamic features fully, including damped voltage oscillations deliberately applied by the high-voltage power supply.

Fig. 4 ▸ shows preliminary Fizeau measurement of Mirror 1’s dynamic radius of curvature as a function of time *t*, after applying high voltage to all piezo electrodes (*t* = 0). In this representative scan, performed to validate our data-acquisition and analysis algorithms, a relatively slowly varying damped sinusoidal voltage was applied immediately after a large voltage impulse (1000 V). In this scan, and others with different voltage impulses, all expected features were clearly resolved by the Fizeau interferometer. This proved that the instrument has sufficient temporal and topographical sensitivity to record precisely the dynamic changes in the mirror’s radius of curvature.

Curvature data from the Fizeau were cross-checked for accuracy against Diamond-NOM measurements. In all cases, changes in the inverse radius of curvature were proportional to the voltage delivered by the HV power supply, as predicted by previous theoretical (Susini *et al.*, 1996 ▸) and experimental studies (Signorato *et al.*, 1998 ▸; Alcock *et al.*, 2015 ▸). Also, no appreciable time lag was observed between voltage application and curvature change.

Having proven the suitability of high-speed Fizeau interferometry to characterize the dynamic bending of bimorph mirrors, we progressed to a systematic study to understand the causes of residual curvature drift.

### 3.1. Fizeau interferometry of a first-generation bimorph (Mirror 1)   

**Figure 5:**
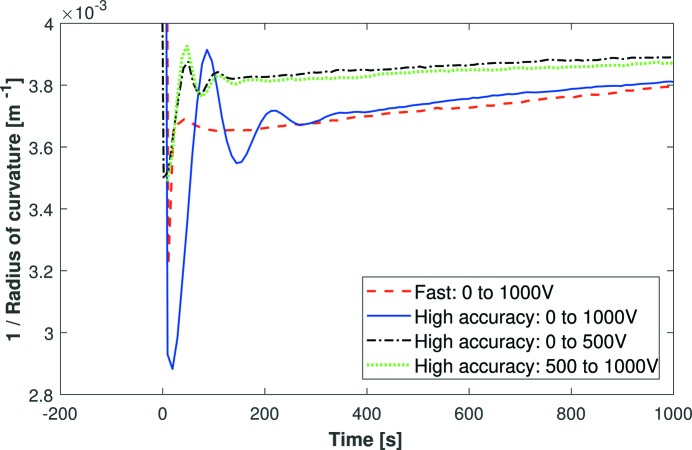
The dynamic radius of curvature of Mirror 1, as measured by a Fizeau interferometer, as a function of time after applying a voltage change of 500 or 1000 V to all piezo electrodes. High-accuracy mode has a voltage slew rate <10 V s^−1^, and deliberately applies damped sinusoidal voltage oscillations after the main impulse. Fast mode has a voltage slew rate of 50 V s^−1^. Oscillations and drift rates in the radius of curvature provide important information about how the mirror responds to voltage, and how the holder and electrical connectors constrain it.

An obsolete (>10 years old) first-generation bimorph with solid electrical connectors and PEEK clamps (Mirror 1 in Table 1 ▸) was monitored by the Fizeau interferometer in response to a variety of applied voltage changes, including sinusoidal oscillations of different amplitude and frequency (Fig. 5 ▸). Since the change in the inverse radius of curvature is linearly proportional to the applied voltage, a constant vertical offset can be added to the inverse radius data without distorting the bend range. This offset enables comparison between mirrors with different pre-polished radii or bending ranges, or the same mirror bent to several curvatures. In Fig. 5 ▸, a vertical offset has been added to the 0 to 500 V curve to aid comparison with the other curves where the mirror’s final voltage is 1000 V. A small offset is retained to aid visibility between the curves.

Several conclusions can be drawn from the data. Firstly, the curvature drift rate (*i.e.* the long-term trend after the initial oscillations) for the 0 to 1000 V change (red dashed and blue solid curves) is independent of the power supply mode (fast or high accuracy). Secondly, as anticipated, the radius drifts more rapidly (*i.e.* a steeper gradient) after a 1000 V change (red dashed and blue solid curves) than after a 500 V change (green dotted and black dot–dashed curves). Finally, the rates of drift for both Δ*V* = 500 V curves (0 to 500 V and 500 to 1000 V) are very similar. This indicates that the relative change in voltage, rather than its absolute value, influences the magnitude of the drift. Over 1 h, the radius of curvature for Mirror 1 drifted by >2% and >5% for 500 V and 1000 V changes, respectively. However, from measurements of a single mirror it is not possible to determine uniquely whether the holder, the solid electrical connectors or intrinsic piezo creep is the prime cause of the observed curvature drift.

### 3.2. Fizeau interferometry of a first-generation bimorph with flexible electrical wires (Mirror 2)   

To isolate and better understand the factors which influence the dynamic bending of bimorphs, we progressed to Mirror 2 (see Table 1 ▸) which is a more recent first-generation super-polished (EEM) bimorph. Like Mirror 1, it is reasonably thin (<10 mm) and has a similar non-kinematic holder. Leaf springs push the substrate onto four PEEK clamps at its corners. A major difference is that Mirror 2 has flexible wires to provide voltages to the piezo electrodes, rather than the rigid connectors of Mirror 1.

**Figure 6:**
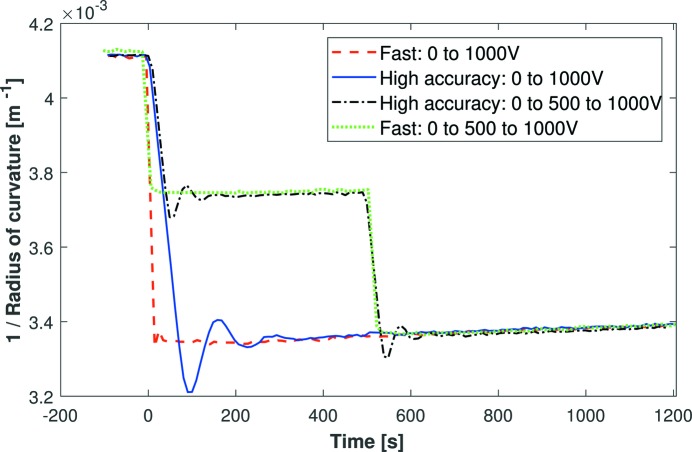
Fizeau interferometry capturing dynamic changes in the inverse radius of curvature of Mirror 2 (a super-polished first-generation bimorph) after a 1000 V impulse. The solid and dashed curves show curvature data with the HV power supply operating with different slew rates (high accuracy <10 V s^−1^ and fast = 50 V s^−1^). An intermediate jump to 500 V, or a higher voltage slew rate, did not change the decay rate of the radius of curvature.

Fig. 6 ▸ shows Mirror 2 being driven from 0 to 1000 V using various voltage profiles provided by the HV-ADAPTOS power supply’s programmable modes. Firstly, all four curves have identical curvature decay rates: this reinforces the conclusion that changing the operational mode does not shorten the stabilization period. Secondly, temporarily driving to an intermediate voltage step, in this case 500 V, does not change the decay rate. Overall, for Δ*V* = 1000 V, the curvature of Mirror 2 drifts by ∼3% over 1 h, which is an improvement compared with ∼5% for Mirror 1. This confirms that the upgraded flexible wire connectors are beneficial compared with the rigid connectors.

Interestingly, Mirrors 1 and 2 both drift in the opposite direction with respect to the bending induced by the voltage impulse. This effect was observed consistently for Mirrors 1 and 2 for a range of positive and negative voltage changes. For example, after a positive voltage change, the mirror became less concave but then gradually became slightly more concave over time. The reverse occurs for a negative voltage change. As piezo creep is expected to occur in the same direction as the voltage impulse, the observed drift in the opposite direction can be attributed to strain from the opto-mechanical holder, which resists the action of piezo bending.

### 3.3. Fizeau interferometry of a second-generation bimorph in an improved holder (Mirror 3)   

**Figure 7:**
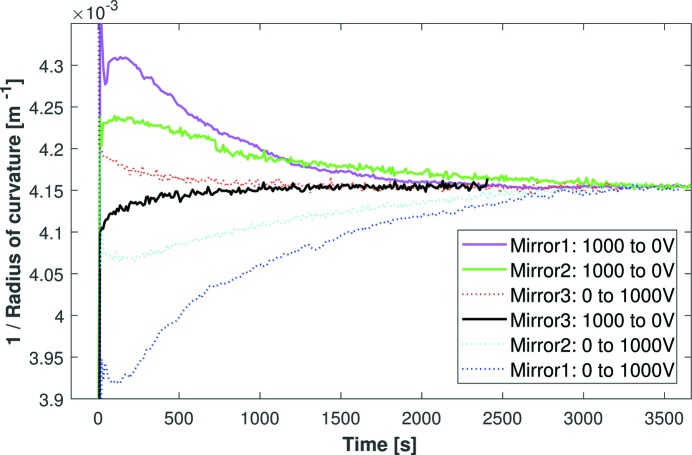
The stabilization of curvature for the three bimorph mirrors in the hour after applying +1000 V (dotted curves) or −1000 V (solid curves) to all piezos. A substantial improvement in the stability of the radius is observed for Mirror 3, a second-generation bimorph with flexible wire connections and clamped into an optimized opto-mechanical holder. Note that the direction of curvature drift of Mirror 3 is in the opposite sense to the two other mirrors

Having identified that the old-fashioned holders and solid electrical connectors were causing each mirror’s curvature to drift, the next step was to utilize an improved opto-mechanical holder (as described in Section 2.3) and wire-based electrical connections for a new second-generation bimorph with a super-polished substrate. Fig. 7 ▸ shows the Fizeau measurement of Mirrors 1, 2 and 3 after applying a voltage change of 0 to 1000 V or 1000 to 0 V. As we are mostly interested in relative changes in curvature, each inverse radius curve is vertically offset to approach the same asymptotic value as *t* → ∞. Mirror 3 has subsequently been installed, along with its KB partner, on the Micro-focus Macromolecular Crystallography (I24) beamline at Diamond.

As hoped, the curvature of Mirror 3 drifts significantly less than the other two mirrors. The magnitude of drift for Mirror 1 is ∼2.9 or ∼2.1 times larger than Mirror 2 for positive or negative voltage changes, respectively. A proportion of this improvement can probably be attributed to the use of flexible wires rather than solid electrical connectors. Similarly, Mirror 3 drifts by a factor of 1.6 less than Mirror 2, which is probably due to the improved kinematic holder. Overall, Mirror 3 drifts 4.6 or 3.4 times less than Mirror 1 for the two voltage directions. Drifts for positive or negative voltage changes are symmetric for Mirrors 2 and 3, but asymmetric for Mirror 1 in Fig. 7 ▸. A possible explanation for this asymmetry is that the solid electrical connectors of Mirror 1 are in a neutral position at 0 V, but as the voltage is increased, and the substrate bends, the solid electrical connectors become strained and apply a resisting force to the substrate. The flexible wires of Mirrors 2 and 3 cannot oppose bending, and hence the corresponding curvature drifts are symmetric. A final important point to note about Fig. 7 ▸ is that the curvature drift for Mirror 3 is in the opposite direction with respect to the other two mirrors. This indicates that Mirrors 1 and 2 are dominated by resistive forces from the holders and/or solid electrical connectors (*i.e.* the holder is trying to pull the mirror’s shape back to its neutral 0 V position). Conversely, these factors have been minimized for Mirror 3, and the small amount of residual curvature drift is in the same direction as the bending, as expected for piezo creep.

### 3.4. Reducing the voltage application period   

**Figure 8:**
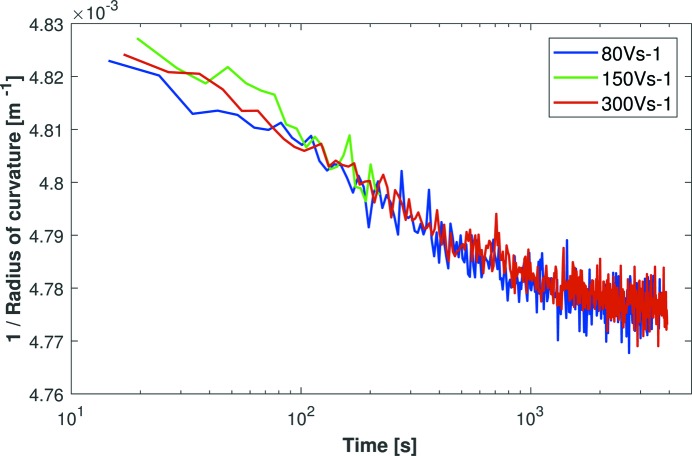
The drift in the inverse radius of curvature of Mirror 3, as measured by a Fizeau interferometer, is independent of the power supply’s voltage slew rate (80, 150 or 300 V s^−1^). This means that large (1000 V) voltage changes can be applied to a bimorph mirror in a matter of seconds without compromising the rate of the mirror’s subsequent curvature drift. A logarithmic change in the mirror’s curvature as a function of time further strengthens the argument that the remaining drift is due to piezo creep.

Can the time taken to apply voltages be reduced by simply increasing the voltage slew rate? If so, does it affect the rate of drift in the mirror’s curvature? To investigate these questions, a 1000 V voltage change was applied to Mirror 3 from the HV-ADAPTOS power supply using faster slew rates of 80, 150 or 300 V s^−1^. Fig. 8 ▸ confirms that the residual curvature drift is independent of the voltage slew rate. It is therefore preferable to use a high slew rate since this significantly reduces the time required to apply voltages, whilst not influencing subsequent curvature drift. For example, in the most extreme case shown in Fig. 8 ▸, a 1000 V impulse was applied in only ∼3 s, which is 30 times faster than the typical default setting of ∼10 V s^−1^.

Interestingly, with time plotted on a logarithmic scale in Fig. 8 ▸, the drift in inverse radius of curvature follows a logarithmic relationship, particularly for *t* < 1000 s. This matches perfectly the expectation for piezo creep. A logarithmic drift relationship was not found for Mirrors 1 or 2, where the curvature drift was dominated by mechanical strain from the opto-mechanical holders and/or electrode connectors.

### 3.5. Reducing the stabilization time: creep compensation   

As shown in Fig. 7 ▸, the design changes made to Mirror 3 have successfully improved its curvature drift performance compared with the other two older mirrors. But can the remaining drift, attributed to piezo creep, be further reduced, thereby minimizing the curvature stabilization period? Each mirror has its own unique constant of proportionality, *c*, which describes how it bends in response to a given voltage. Using this parameter and the initial radius of curvature (*R*_1_), the final radius (*R*_2_) can be calculated after applying a voltage change Δ*V*,
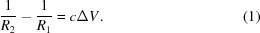



For Mirror 3, *c* = −5.8682 × 10^−7^ V^−1^ m^−1^ was obtained by measuring the change in radius over the mirror’s full bending range. Since the applied voltage is proportional to the inverse radius, one can counteract the change in radius due to piezo creep Δ*R*_creep_(*t*) by applying a time-varying compensating voltage profile *V*_comp_(*t*) immediately after the initial voltage ramp,
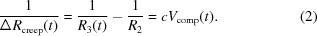



Two methods can be employed to find the optimum compensating voltages. Firstly, one can monitor the dynamic radius of curvature *R*_3_(*t*) and manually apply voltage corrections in real time. Alternatively, one can measure the curvature drift without any compensating voltages, calculate the necessary compensating voltage profile as a function of time *V*_comp_(*t*) using equation (2) and apply these voltages during a re-run of the experiment. The control software of the HV-ADAPTOS power supply was reprogrammed to enable a list of correction voltages as a function of time to be read in from file and applied automatically immediately after the main voltage ramp.

**Figure 9:**
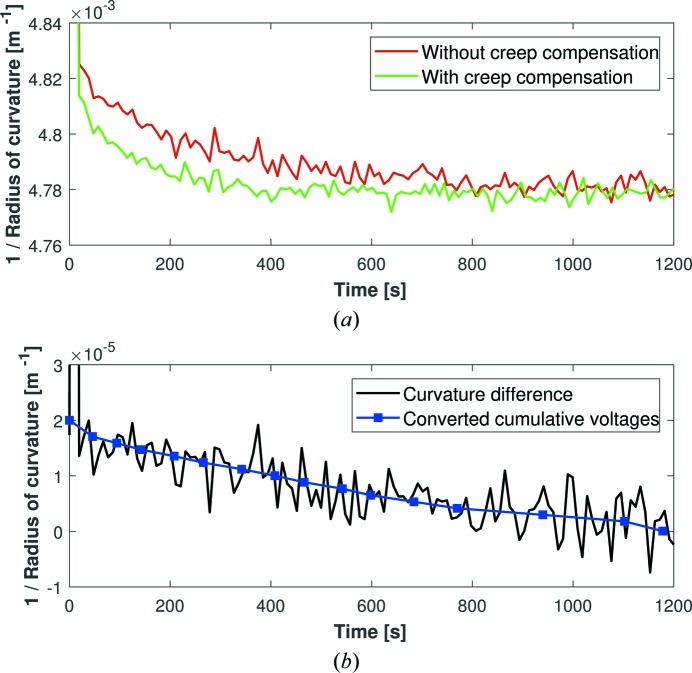
(*a*) The dynamic radius of curvature of Mirror 3, as measured by a Fizeau interferometer, with (green curve) and without (red curve) corrective voltages uniformly applied to all electrodes to compensate for piezo creep following a 1000 V impulse. Using creep compensation, we have shown for the first time that a synchrotron X-ray bimorph mirror can be bent and stabilized to <1% of any given curvature within <10 s. (*b*) The black line shows the measured change in curvature [the difference between the two curves in panel (*a*)] as a function of time, compared with the output of equation (2) where applied creep-compensation voltages are converted into predicted curvature changes (blue squares).

To investigate the first compensation method, correction voltages were applied manually to Mirror 3 in real time using feedback of the mirror’s curvature from the Fizeau interferometer. Fig. 9 ▸(*a*) shows the curvature drift for two runs, one with and one without creep compensation applied immediately after the main voltage ramp of 1000 V. Overall, a cumulative total of ∼50 V was required to compensate for piezo creep, corresponding to only a 5% correction of the 1000 V change. This process successfully reduced the magnitude of piezo creep, and shows for the first time that a synchrotron X-ray bimorph mirror can be bent and stabilized to <1% of a given curvature within <10 s.

To investigate the second compensation scheme, the solid black line in Fig. 9 ▸(*b*) shows the change in the inverse radius of curvature made by creep compensation [*i.e.* the difference between the two curves in Fig. 9 ▸(*a*)]. The cumulative voltage, applied uniformly to all piezos as a function of time to correct piezo creep, was converted into a corresponding curvature change using equation (2). In Fig. 9 ▸(*b*), excellent agreement is observed between the calculated curvature change (blue squares) and the measured change in curvature (solid black line), thereby proving the validity of equation (2). This shows that in future, rather than manually tweaking the compensating voltages based on real-time metrology feedback, it would be more efficient to use equation (2) to calculate the correction voltages directly based on an initial measurement of piezo creep. Importantly, these operations were repeatable, meaning that once suitable voltages are found to compensate creep for a given voltage ramp, corrections can be applied from a look-up table without the need for continual metrology monitoring.

With piezo creep compensation applied, the curvature of Mirror 3 stabilized to <0.5% in 50 s, <0.25% in 130 s and <0.1% in 220 s. These values could be further improved by iterative optimization of the corrective voltages, or by directly calculating the correction voltages using equation (2). Therefore, even including the ∼3 s to apply the 1000 V ramp (which induces a major change in the X-ray focusing properties of the mirror), we have achieved our goal of bending and stabilizing the radius of curvature to <1% in <10 s, and thus provide the first demonstration of high-speed operation of bimorph deformable X-ray mirrors. In principle, a similar voltage-compensation routine could be used to reduce the larger drifts of Mirrors 1 and 2 induced by their holders. However, in practice this could be highly problematic due to the complex interaction between mirror, holder and electrical connectors.

## 4. Conclusions   

We have performed the first visible-light metrology investigation of the dynamic bending behaviour of piezoelectric bimorph deformable micro-focusing X-ray mirrors for synchrotron and XFEL sources. Acquisition scripts and analysis software have been developed to show that high-speed Fizeau interferometry is a suitable technique for observing rapid changes in the optical surface in response to applying voltage changes to the piezo electrodes. The main causes of curvature drift have been identified, and we have shown how such effects can be reduced significantly by using flexible electrical wires, an improved opto-mechanical holder and a programmable high-voltage power supply to increase the voltage slew rate and compensate for piezo creep. We have demonstrated that major changes can be reliably made to the optical profile of bimorphs in just a few seconds. Such actions can be repeated indefinitely since the piezoelectric actuators do not dissipate power during operation. It is hoped that such knowledge will benefit many beamlines, eventually leading to the rapid stabilization of the size, shape and position of X-ray beams reflected by bimorphs.

Part II of this study (Alcock *et al.*, 2019 ▸) builds upon the ex situ results and shows how Mirror 3 installed with its KB partner on the I24 beamline at Diamond can rapidly provide a range of sizes and shapes of X-ray beams.

## Figures and Tables

**Table 1:** Parameters of the three microfocusing bimorph deformable mirrors investigated in this ex situ metrology study of dynamic bending behaviour

	Mirror 1	Mirror 2	Mirror 3
Production year	2006	2009	2016
Polishing	Mechanical	EEM	EEM
Length (mm)	150	150	240
Width (mm)	35	40	34
Number of piezos	8	8	16
Generation	First	First	Second
Holder	Non-kinematic	Non-kinematic	Kinematic
Electrical connectors	Rigid	Wires	Wires
